# Modifiable cardiovascular disease risk factors as predictors of dementia death: pooling of ten general population-based cohort studies

**DOI:** 10.1186/1477-5751-13-8

**Published:** 2014-05-23

**Authors:** G David Batty, Tom C Russ, John M Starr, Emmanuel Stamatakis, Mika Kivimäki

**Affiliations:** 1 Department of Epidemiology and Public Health, University College, 1-19 Torrington Place, London, UK; 2 Centre for Cognitive Ageing & Cognitive Epidemiology, University of Edinburgh, Edinburgh, UK; 3 Alzheimer Scotland Dementia Research Centre, University of Edinburgh, Edinburgh, UK; 4 Scottish Dementia Clinical Research Network, NHS Scotland, Edinburgh, UK; 5 Division of Psychiatry, University of Edinburgh, Edinburgh, UK

## Abstract

**Background:**

With drug treatment for dementia being of limited effectiveness, the role of primary prevention, in particular the predictive value of modifiable cardiovascular disease risk factors, may warrant exploration. The evidence base is, however, characterised by discordant findings and is modest in size. Accordingly, we examined the association of modifiable cardiovascular disease risk factors with dementia death.

**Design and methods:**

We pooled raw data from 10 UK general population-based prospective cohort studies within the context of an individual participant meta-analysis.

**Results:**

A total of 103,764 men and women were followed up for a mean of 8 years giving rise to 443 dementia-related deaths and 2612 cardiovascular disease deaths. Cardiovascular disease mortality was, as anticipated, associated with the full range of risk factors under study, including raised blood pressure, smoking, diabetes, physical inactivity. By contrast, dementia death was related to very few of the cardiovascular disease risk factors: of those classified as modifiable, only smoking was associated with a raised risk and higher levels of non-HDL with a lower risk.

**Conclusions:**

In the present individual participant meta-analysis, there was limited evidence that cardiovascular disease risk factors were related to dementia death.

## Background

The present and future social, economic, and disease burden exacted by dementia is considerable and well documented. That drug treatment, even when implemented in the early stages of the illness, is only symptomatic and of limited effectiveness raises the need to identify primary risk factors for dementia. If modifiable environmental indices are shown to predict dementia, this could have implications for control of the disease.

In particular, it has been posited that cardiovascular disease (CVD) risk factors, many of which are modifiable, are linked to dementia development. The notion that CVD and dementia or cognitive impairment may have a shared aetiology was first advanced over four decades ago [1]. However, in a recent consensus statement, an expert panel concluded that for Alzheimer’s Disease, which represents up to 70% of all dementia cases [2], there is insufficient evidence with which to form firm conclusions about the influence of any modifiable environmental characteristic, including CVD risk factors [3].

Accordingly, we examined the association of a range of established CVD risk factors with dementia death. To do so, as we have done in different contexts [4-7], we present a meta-analysis of individual-participant data from ten UK population-based cohort studies. Taking this approach involves the pooling of raw data. In contrast to the more traditional literature-based meta-analysis in which investigators may have to exclude publications that do not present results in a standard manner, the possibility of publication bias is reduced in an individual participant meta-analysis. Additionally, a literature based meta-analysis cannot provide a consistent approach to statistical control for plausible covariates. While the individual participant meta-analysis approach has been taken for physiological risk factors in relation to somatic disease outcomes [8,9], it is much less common in the field of psychiatry. To our knowledge, this is the largest study to date to explore the link between individual CVD risk factors and dementia death and the first individual participant meta-analysis. For the purposes of comparison, we also report on the relation of the same collection of risk factors with CVD mortality.

## Methods

The Health Surveys for England (conducted 1998, 1999, 2003–6, and 2008) [10] and the Scottish Health Surveys (1995, 1998, and 2003) [11] are UK-representative, repeat, cross-sectional, independent studies with on-going mortality surveillance of their study members. Participants gave informed consent; ethical approval was obtained from the London Research Ethics Council. Participants completed a questionnaire and took part in clinical examination. During both, CVD risk factors were assessed using standard operating protocols as have been described in detail [5,7].

Participants were linked to national mortality registers. Causes of death (up to ten) recorded on certificates were coded using the International Classification of Diseases, Ninth (ICD-9) [12] and Tenth (ICD-10) [13] revisions. Any mention of dementia death was identified using codes 290.0 to 290.4, 294.9, 331.0 to 331.2, and 331.9 for ICD-9 and F00, F01, F03, F09, G30 and G31 for ICD-10. We have previously shown that in a cohort of Scottish individuals with psychiatrist-confirmed dementia, almost three quarters of those who subsequently died had dementia recorded on their death certificate [14]. This suggests that the use of dementia deaths data has some validity.

### Statistical analyses

Having ascertained that the proportional hazards assumption had not been violated, we then used Cox proportional hazards models [15] to compute study-specific hazard ratios with accompanying 95% confidence intervals for the association between each CVD risk factor and dementia death. Preliminary results suggested that there was no consistent evidence for differential risk factor-dementia effects by gender; data for men and women were therefore pooled with hazard ratios sex-adjusted.

The study-specific effect estimates and their standard errors were pooled using a random effects meta-analysis. In contrast to a fixed-effects meta-analysis, a random-effects approach allows for variation in observed risk factor–disease estimates across studies which occur because of real differences in the association in each study and/or sampling variability. We chose *a priori* a random-effects technique for two reasons. First, many of the CVD risk factors featured in the present report (raised blood pressure, obesity, physical activity, smoking and so on) have been the subject of primary care-based modification during the period of follow-up of the included studies. Depending on the era of the study in question, this may have had an impact on the risk factor–disease associations under scrutiny. Second, there have now been a series of papers from this collaboration in which the outcomes of interest have been the same as the present analyses–CVD [5,7] and dementia [4-6]–although the predictor variables have differed. That these existing results suggest evidence of heterogeneity across studies, provided us with a second reason to utilise the random-effects approach.

Heterogeneity in the effect estimates between studies was examined by computing an I^2^ statistic, which indicates the proportion of the total variation in the estimates that is due to between-studies variation. Calendar time (months) was the time scale. We censored study members at death or the end of follow-up (15th February 2011), whichever came first.

## Results

From an initial sample of 122,060 participants, 18,296 did not consent to mortality record linkage or, if they did, had missing survival or mortality data, giving a maximum analytic sample of 103,764 (55% female, mean age 47.3 years, SD = 18.1, range = 16-102). Of 7933 deaths occurring during a mean (SD) follow up of 7.9 (3.8) years, 443 were dementia-related and 2612 were cardiovascular disease-related. With dementia as the outcome of interest, the I^2^ statistic varied between 0% and 76.4% depending on the CVD risk factor in question. This provided *post hoc* justification of our random-effects approach to the meta-analysis.

Table 1 depicts the association of fourteen CVD risk factors with dementia death and CVD death risk. Importantly, known risk factor–CVD relationships were all replicated in the present analyses. Thus, conventionally unfavourable levels of blood pressure and total cholesterol, high body mass index, smoking, diabetes and low physical activity were associated with higher CVD death rates, although effect of systolic hypertension did not reach statistical significance.

**Table 1:** Age- and gender-adjusted hazard ratios (95% confidence intervals) for the relation of individual cardiovascular disease risk factors with dementia death and cardiovascular disease death: individual participant meta-analysis of ten general population-based cohort studies

**Risk factor**	**Comparison**	**Cardiovascular disease**				**Dementia**			
		**Deaths**	**N**^**k**^	**HR (95% CI)**	**P-value**	**Deaths**	**N**^**k**^	**HR (95% CI)**	**P-value**
Age	Per 5 yr increase	2612	103764	1.78 (1.74, 1.82)	<0.001	443	103764	2.42 (2.29, 2.56)	<0.001
Gender	Female vs. male	2612	103764	0.60 (0.53, 0.68)	<0.001	443	103764	0.78 (0.65, 0.95)	0.01
Marital status	See footnote^a^	990	61171	1.34 (1.00, 1.80)	0.049	156	61171	0.94 (0.58, 1.53)	0.81
Occupational social class	Non-manual vs. manual	2486	97455	1.36 (1.18, 1.57)	<0.001	424	97455	1.11 (0.86, 1.43)	0.42
Educational attainment	Left school ≤ 15 yr vs. older	2610	103680	1.38 (1.25, 1.52)	<0.001	443	103680	1.18 (0.95, 1.45)	0.13
Hypertension	See footnote^b^	1600	63989	2.10 (1.83, 2.41)	<0.001	283	61751	0.96 (0.71, 1.30)	0.79
Systolic blood pressure	See footnote^c^	1600	63989	1.08 (0.90, 1.30)	0.39	283	61751	0.87 (0.68, 1.11)	0.27
Diastolic blood pressure	See footnote^d^	1600	63989	1.50 (1.31, 1.71)	<0.001	283	61751	1.02 (0.69, 1.50)	0.92
Uncontrolled hypertension	See footnote^e^	803	46410	2.55 (2.08, 3.12)	<0.001	128	44626	0.88 (0.60, 1.29)	0.52
Smoking status	Current smoker vs. other	2606	103282	1.86 (1.62, 2.15)	<0.001	443	103282	1.75 (1.33, 2.29)	<0.001
Current drinker	Current vs. never/former^f^	2610	103072	0.77 (0.64, 0.94)	0.01	443	103072	0.85 (0.67, 1.07)	0.17
High total cholesterol	See footnote^g^	1424	55355	1.30 (1.04, 1.62)	0.02	233	53375	0.88 (0.67, 1.15)	0.34
Non-HDL cholesterol^h^	Per SD (1.2 mmol/L) rise	1135	49562	1.06 (1.00, 1.19)	0.04	200	42271	0.82 (0.70, 0.96)	0.01
Serum C-reactive protein	Per SD (6.7 mmol/L) rise	1022	39352	1.14 (1.07, 1.21)	<0.001	191	37439	1.03 (0.93, 1.15)	0.56
Body mass index	≥ 30 kg/m^2^ vs. < 30	1952	90978	1.24 (1.12, 1.37)	<0.001	322	90978	0.71 (0.53, 0.97)	0.03
Diabetes status	Yes vs. no^i^	2605	100639	1.97 (1.65, 2.35)	<0.001	441	100639	1.03 (0.73, 1.46)	0.85
Physical inactivity	< 5 sessions/week vs. ≥ 5^j^	2404	95426	1.63 (1.40, 1.89)	<0.001	401	95426	1.17 (0.72, 1.91)	0.52
Longstanding illness	Any vs. none	2612	103742	1.91 (1.74, 2.09)	<0.001	443	103742	1.10 (0.90, 1.36)	0.35

^a^Single, separated, divorced, or widowed vs. married, civil partnership or cohabiting. ^b^Systolic blood pressure ≥ 140 mmHg, diastolic blood pressure ≥ 90 mmHg, or taking antihypertensive treatment (NICE guidance) vs. other. ^c^Systolic blood pressure ≥ 140 mmHg vs other. ^d^Diastolic blood pressure ≥ 90 mmHg vs other. ^e^Despite antihypertensive medication, either systolic blood pressure ≥ 140 mmHg, diastolic blood pressure ≥ 90 mmHg, or both vs. normotensive individuals. ^f^Current drinkers (any amount) compared to never- or ex-drinkers. ^g^Serum total cholesterol ≥ 6.2 mmol/L or on lipid-lowering treatment versus other. ^h^Non-HDL cholesterol (calculated by subtraction of HDL-C from total cholesterol, yielding a measure that encompasses low-, intermediate-, and very-low-density lipoprotein cholesterol). Hazard ratios are per standard deviation increase (disadvantage). ^i^Diabetes denoted by doctor-diagnosed diabetes, longstanding illness (diabetes), HbA_1c_ ≥ 6.0%, and/or diabetes medication. ^j^Fewer than five average weekly sessions of moderate to vigorous physical activity including domestic (walk/domestic 30 mins+, sports/exercise 15 mins+) compared to five or more (UK government recommendations). ^k^Some cohort studies were excluded from specific risk factor analyses owing to the absence of any dementia deaths.

By contrast, dementia was associated with very few of the CVD risk factors. Being male, older, and smoking were shown to confer an increased risk of dementia death, while higher non-HDL cholesterol and obesity were both associated with protection. The result for obesity was surprising. Speculating that this could be ascribed to reverse causality–dementia influencing the risk factor rather than the converse–led us to drop dementia deaths occurring in the first five years of follow-up (‘left’ censoring) and repeat our analyses (a standard analytical procedure in epidemiology). This attenuated the obesity–dementia association to the null (HR; 95% CI: 0.88; 0.62-1.26 based on 214 dementia deaths in 70391 men and women).

Finally, for the four CVD risk factors found to be associated with dementia death in the above age- and gender-adjusted analyses we carried out mutual adjustment. Based on 322 dementia deaths in the 60625 men and women with complete data, and using the same comparators, our results were essentially unchanged: age (2.53; 2.35, 2.73), gender (0.80; 0.64, 1.00), smoking (1.60; 1.15, 2.22) and body mass index (0.72; 0.53, 0.97).

## Discussion

In the present study-to our knowledge, the first meta-analysis in the field-there was limited evidence that CVD risk factors were related to an increased occurrence of dementia death. The few positive results included higher age, male sex, smoking and, surprisingly being non-obese, the last finding disappearing when taking into account reverse causality. That established risk factor–CVD associations were confirmed, gives us some confidence in the results for dementia.

While our dataset has its strengths–its size and the comprehensive set of CVD risk factors variables assessed–it is of course not without its shortcomings. While we had data on a series of health behaviours (smoking, alcohol intake, and exercise) we did not have information on dietary characteristics which may be linked to dementia risk. Additionally, comprising a series of subtypes (Alzheimer’s disease, vascular dementia, AIDS dementia, and so on), dementia is not a single nosological entity. As it is likely that these subtypes do not have a unifying aetiology, using a composite dementia endpoint may mask some subtype-specific associations with CVD risk factors. It is also the case, however, that, with the majority of dementias being Alzheimer’s, our findings are most applicable to this dementia presentation. Finally, our outcome of interest was dementia death, not incidence; as such, our exposures are more distant from dementia event. This notwithstanding, as described, in a separate study [14], we found that, in a cohort of patients diagnosed with probable Alzheimer’s disease, of the 502 who had died after 11 years of follow-up, 72% had their dementia recorded as either a primary or contributing cause on their death certificate. This suggests that using dementia death may approximate dementia incidence.

Taking these results together, there was limited evidence that these CVD risk factors were related to the occurrence of dementia death in the current study.

## Competing interests

The authors declare that they have no competing interests.

## Author contributions

GDB conceived the idea for the present manuscript; ES built the dataset; and TCR carried out the data analyses. GDB, TCR and MK interpreted the results, and GDB wrote the first draft of the manuscript on which all authors provided comments. All authors read and approved the final manuscript for submission.
